# Correction to “H_2_O(aq) Does Not Exist: Critique of a Proof-of-Concept Derivation”

**DOI:** 10.1021/acs.jchemed.3c00367

**Published:** 2023-05-17

**Authors:** Thomas L. Neils, Todd P. Silverstein, Stephanie Schaertel

In the Discussion section, the sentence “This value, along with the equilibrium constant expression for acid ionization allowed them to calculate *K*_a_(H_2_O) = 1.0(10^–7-^)/55.3 = 1.8 × 10^–16^, so p*K*_a_(H_2_O) = 15.7” should read “This value, along with the equilibrium constant expression for acid ionization, allowed them to calculate *K*_a_(H_2_O) = (1.0 × 10^–7^)^2^/55.3 = 1.8 × 10^–16^, so p*K*_a_(H_2_O) = 15.7.”

